# Integrated Network Pharmacology and Experimental Validation Approach to Systematically Elucidate the Cardioprotective Mechanisms of Shengmai Injection

**DOI:** 10.1155/cdr/6915907

**Published:** 2026-06-28

**Authors:** Nan Hu, Hongnv Zhang, Zhenghao Wang, Shaodong Zhai, Ruiping Zhang

**Affiliations:** ^1^ Third Hospital of Shanxi Medical University, Shanxi Bethune Hospital, Shanxi Academy of Medical Sciences, Tongji Shanxi Hospital, Taiyuan, China; ^2^ Institute of Medical Technology Research, Shanxi Medical University, Taiyuan, China, sxmu.edu.cn; ^3^ The Radiology Department of Shanxi Provincial People′s Hospital Affiliated to Shanxi Medical University, Taiyuan, China

**Keywords:** cardiomyocyte apoptosis, heart failure, myocardial infarction, network pharmacology, Shengmai injection

## Abstract

Core to advancing the study of traditional Chinese medicine (TCM) mechanisms is the integrated “computational prediction–experimental verification” strategy, which enables the precise identification of therapeutic targets through multilevel technological integration. Within this framework, this study systematically investigates the therapeutic mechanisms of Shengmai injection (SMI) against heart failure following myocardial infarction (MI). Initially, using network pharmacology approaches, we systematically screened TCMSP, BATMAN, and disease databases to identify 237 common targets of SMI and chronic heart failure. Through Cytoscape‐based network topology analysis, we pinpointed 52 core targets, with KEGG pathway enrichment analysis revealing the PI3K‐AKT signaling pathway as the crucial pathway. Subsequently, molecular docking technology demonstrated strong binding affinity (calculated affinity: −6.171 kcal/mol) between vitamin E (an active constituent by network pharmacology analysis) and the core target AKT1. At the experimental validation level, we established a mouse MI model by ligating the left anterior descending coronary artery, with continuous administration of SMI for 4 weeks. Experimental results showed that SMI significantly improved cardiac function of chronic heart failure, reduced myocardial fibrosis, inhibited cardiomyocyte apoptosis, and promoted macrophage polarization toward the reparative M2 phenotype. In the H9c2 cell model, SMI effectively improves mitochondrial function of cardiac cells induced by H_2_O_2_ and inhibits reactive oxygen species accumulation and mitochondrial membrane potential collapse. Mechanistic studies revealed that SMI regulates mitochondrial dysfunction by activating the AKT/Bcl‐2 signaling axis, upregulating Bcl‐2 expression, and inhibiting Bax. Through systematic methodological integration, this study not only elucidates the mechanism by which SMI inhibits mitochondrial dysfunction and macrophage polarization and attenuates ventricular remodeling through the AKT/Bcl‐2 pathway but also provides a referential multidisciplinary research paradigm for investigating the mechanisms of TCM formulations.

## 1. Introduction

Traditional Chinese medicine (TCM), leveraging its network pharmacology approach, has emerged as a significant therapeutic strategy for complex diseases. Characterized by its multicomponent, multitarget integrated actions, this approach aligns closely with the holistic philosophy of TCM and enables systematic intervention in the multifaceted mechanisms of diseases. Research based on network pharmacology provides a systematic framework for understanding the therapeutic efficacy of TCM in treating complex diseases by identifying core active components, predicting target interactions, and elucidating mechanistic pathways [1]. In specific areas such as cardiovascular diseases, network pharmacology has been applied to predict the mechanisms of action, toxicity, and metabolic characteristics of TCM, opening new avenues for precision medicine in cardiovascular care [2]. These research advances highlight the distinctive advantages of TCM in treating complex diseases through its “multicomponent, multitarget, multipathway” approach, significantly advancing the modernization of TCM [3]. Therefore, network pharmacology provides an innovative strategy for TCM to address complex diseases, offering not only a scientific basis for clinical application but also laying a solid foundation for its global dissemination. By capitalizing on the holistic and systematic nature of TCM, network pharmacology has established a new paradigm for transforming traditional experience‐based medicine into an evidence‐based medical system, thereby accelerating the development of new TCM‐based therapies and optimizing modern drug discovery strategies [4].

Heart failure (HF), the end‐stage manifestation of cardiovascular diseases, remains a significant and escalating global health challenge, contributing to exceedingly high rates of morbidity, mortality, and healthcare burden [5]. A primary antecedent to HF development is myocardial infarction (MI), which serves as a frequent initiating event in its pathogenesis [6, 7]. A complex and maladaptive process known as pathological ventricular remodeling ensues after MI. This remodeling is characterized by the irreversible loss of functional cardiomyocytes through cell death, excessive deposition of extracellular matrix proteins leading to interstitial and replacement fibrosis, progressive chamber dilation, and alterations in ventricular geometry and wall thinning [8, 9]. Crucially, while the initial ischemic insult triggers acute cardiomyocyte necrosis within the infarct core, a sustained wave of cardiomyocyte apoptosis, particularly prevalent in the vulnerable peri‐infarct region or border zone surrounding the necrotic core, constitutes a major pathological driver of the ongoing remodeling process and the subsequent progression towards overt HF [10, 11].

Shengmai injection (SMI) stands as an exemplary success story of TCM in treating cardiovascular diseases. SMI originates from the classical Shengmai formula (SMF), which comprises three botanical components: *Panax ginseng* C.A.Mey. (Araliaceae), *Ophiopogon japonicus* (Thunb.) Ker Gawl. (Asparagaceae), and *Schisandra chinensis* (Turcz.) Baill. (Schisandraceae) [12, 13]. Clinically, this formulation is primarily employed in managing cardiovascular and cerebrovascular diseases characterized by Qi‐Yin deficiency syndrome [13–15]. Both preclinical and clinical investigations have substantiated its multifaceted therapeutic properties, including antioxidant, anti‐inflammatory, and microcirculation‐enhancing effects [16–18]. However, its specific effects on chronic post‐MI cardiomyocyte apoptosis and the precise mechanism underlying its benefits in post‐MI HF require further investigation.

While current guideline‐directed medical therapies (GDMTs) represent the cornerstone of post‐MI management, they demonstrably offer incomplete protection against the insidious progression of pathological ventricular remodeling [19–21]. Although these interventions significantly improve survival and reduce major adverse cardiac events by mitigating hemodynamic stress, neurohormonal activation, and initial infarct expansion, they fail to fully arrest the underlying cellular and molecular drivers of chronic remodeling. Critically, sustained cardiomyocyte apoptosis, particularly within the vulnerable border zone, and the consequent maladaptive fibrotic response often persist despite optimal application of these therapies. This therapeutic gap—the inability to completely halt ongoing cardiomyocyte loss and extracellular matrix dysregulation—directly contributes to the residual risk of progressive ventricular dilation, systolic and diastolic dysfunction, and the eventual transition to overt HF observed in a significant proportion of post‐MI patients, highlighting the need for novel strategies.

Although numerous studies have investigated the therapeutic mechanisms of SMI in MI, a systematic review and further validation of its mechanisms using network pharmacology approaches remain lacking. In light of this, building upon extensive preliminary research data, this study employs network pharmacology to further elucidate and validate the effective targets and mechanisms of SMI. This work not only synthesizes and summarizes existing research findings but also provides valuable references for the precise clinical application of TCM.

## 2. Materials and Methods

### 2.1. Materials

The cell counting kit‐8 (CCK‐8) and calcein/propidium iodide (PI) live/dead assay kits were obtained from Beyotime Biotechnology (Shanghai, China). JC‐1 (HY‐15534) was purchased from MedChemExpress (Shanghai, China). DCFH‐DA (S0033S) was purchased from Beyotime Biotechnology (Shanghai, China). The Annexin V‐FITC Apoptosis Detection Kit was obtained from Solarbio Science and Technology (Beijing, China). Antiphosphorylated AKT (Ser473) (R381555) and anti‐AKT (R380617) were purchased from Zen‐Bioscience Co. Ltd. (Chengdu, China). Anti‐GAPDH was purchased from ABclonal (Wuhan, China). The SMI (Batch Number: 24042601) used in this study was supplied by Yisheng Pharmaceutical Co. Ltd. (Jilin, China) (National Drug Approval Number: Z22026097). According to the quality control test report provided by the manufacturer, the product contains the following major active compounds: ginsenosides Rg1 and Re at a concentration of 0.18 mg/mL, ginsenoside Rb1 at 0.20 mg/mL, and schizandrin at 10.5 *μ*g/mL.

### 2.2. Methods

#### 2.2.1. Animal Model and Design

Male mice (8 weeks old) were purchased from Huafukang Biotechnology Co. Ltd. (Beijing, China). Mice underwent permanent left anterior descending (LAD) ligation under anesthesia to induce MI. The sham group underwent thoracotomy without coronary artery ligation. The experimental groups randomly assigned into four groups (*n* = 4/group): MI, MI + SMI (2 mL/kg), MI + SMI (4 mL/kg), and MI + SMI (8 mL/kg). Postoperative randomization. SMI was diluted with 0.9% physiological saline and administered intraperitoneal injection (ip) daily for 4 weeks, starting 24 h post‐MI.

#### 2.2.2. Cardiac Function Assessment

Transthoracic echocardiography was performed on anesthetized mice (induced with 3% isoflurane) positioned supine on a thermostatically controlled platform (37°C). Cardiac function in the mice was assessed by transthoracic echocardiography performed using an ultrahigh‐frequency photoacoustic ultrasound imaging system (Vevo LAZR‐X, Fujifilm VisualSonics). The mice were anesthetized with isoflurane. Left ventricular (LV) parameters were obtained from two‐dimensional (2D) short‐axis views acquired at the papillary muscle level, guided by M‐mode imaging. The following key functional and structural indices were quantified: LV ejection fraction (EF%), LV fractional shortening (FS%), LV end‐diastolic volume (LV Vol; d), and LV end‐systolic volume (LV Vol; s). All animal experimental procedures were approved by the Shanxi Bethune Hospital Animal Care and Use Committee (SBQKL‐2021‐040) and were conducted in strict accordance with the National Institutes of Health (NIH) Guide for the Care and Use of Laboratory Animals (NIH Publication No. 85‐23, revised 1985). All efforts were made to minimize animal suffering.

#### 2.2.3. Histological Staining

Histological analysis of the heart was performed using hematoxylin and eosin (H&E), Masson′s trichrome, and picrosirius red staining. Tissues were fixed in 4% paraformaldehyde, processed through graded alcohols and xylene, embedded in paraffin, and sectioned at 5 *μ*m thickness. Following deparaffinization in xylene and rehydration through a graded ethanol series, the sections were stained according to standard H&E, Masson′s trichrome, and picrosirius red protocols. H&E staining was performed to determine the safety of the long‐term medication in the liver, spleen, lungs, and kidneys.

#### 2.2.4. Apoptosis Detection

Apoptosis in the cardiac tissue was assessed by TUNEL staining according to the manufacturer′s protocol (APExBIO, Houston, United States). Paraffin‐embedded heart sections (5 *μ*m) were deparaffinized, rehydrated, and subjected to antigen retrieval. After fixing in 4% paraformaldehyde, the sections were permeabilized with 0.2% Triton X‐100 (10 min) and incubated with the TUNEL reaction mixture. Nuclei were counterstained with DAPI. Apoptotic cells were identified by colocalization of TUNEL‐positive signals (green fluorescence) and nuclear staining.

For H9c2 cells, we induced oxidative stress injury using 100 *μ*M H_2_O_2_ (final concentration in serum‐free DMEM), simultaneously treated with SMI, and incubated for 4 h, after which the medium was removed. Apoptosis assay was assessed by staining the cells with Annexin V‐FITC and PI for 15 min at room temperature (RT) in the dark, followed by immediate analysis using a flow cytometer. Cells (1 × 10^6^/mL) were harvested gently using EDTA‐free trypsin, washed twice with cold phosphate‐buffered saline (PBS), resuspended in buffer to preserve membrane integrity, and quantified using the FlowJo software.

#### 2.2.5. Immunofluorescence Staining

The sections were rehydrated in PBS and subjected to antigen retrieval. Sections were permeabilized with 0.2% Triton X‐100 in PBS for 15 min at RT and then blocked for 1 h at RT with a solution containing 5% normal serum and 1% bovine serum albumin (BSA) in PBS. Primary antibodies (anti‐inducible nitric oxide synthase [iNOS] and anti‐CD206) were applied simultaneously and incubated overnight at 4°C in a humidified chamber. Species‐specific secondary antibodies conjugated to spectrally distinct fluorophores (Alexa Fluor 555 for iNOS and Alexa Fluor 488 for CD206) were added and incubated for 1 h at RT in the dark. Nuclei are counterstained with DAPI (1 *μ*g/mL in PBS) for 5 min at RT. Slides were washed briefly in PBS, excess liquid was carefully removed, and sections were coverslipped and acquired fluorescence micrographs.

#### 2.2.6. Cytotoxicity Assay

Seeded H9c2 cells into 96‐well plates at a density of 5 × 10^3^ cells/well in 100 *μ*L complete medium. Replace the medium with fresh serum‐free medium containing different concentrations of SMI (0–100 *μ*L/mL) and incubate for 24 h. Add 10 *μ*L CCK‐8 reagent directly to each well using a multichannel pipette. The plates were gently swirled to ensure homogeneous mixing. The plate was then returned to the incubator for 2 h to protect it from light and prevent reagent degradation. The plate was transferred to a microplate reader, and the optical density (OD) was measured at 490 nm. We also used live/dead cell staining to evaluate cell viability. Cells incubated with the calcein‐AM and PI dye mixture in serum‐free medium at 37°C for 30 min were protected from light and were observed and photographed under a fluorescence microscope.

#### 2.2.7. Membrane Potential Detection

JC‐1 staining was performed by incubating cells (70%–80% confluency) with 10 *μ*g/mL JC‐1 dye in prewarmed serum‐free medium at 37°C for 20 min under light‐protected conditions, followed by two gentle PBS washes. Mitochondrial depolarization was indicated by a fluorescence shift from red JC‐1‐aggregates to green JC‐1‐monomers, detectable by fluorescence microscopy. Data were expressed as the red/green fluorescence intensity ratio using ImageJ and normalized to the controls.

#### 2.2.8. Reactive Oxygen Species Detection

The DCFH‐DA assay was conducted by incubating the cells with 10 *μ*M DCFH‐DA in serum‐free medium at 37°C for 30 min in the dark. After removing the excess dye via two PBS washes, cells were treated with H_2_O_2_ and incubated for 4 h. The fluorescence intensity was quantified immediately using a fluorescence microscope.

#### 2.2.9. Western Blot

The protein was detected by lysing cells in RIPA buffer supplemented with protease/phosphatase inhibitors, followed by quantification using a BCA assay and separation of 50 *μ*g total protein via 10% SDS‐PAGE. Proteins were transferred to PVDF membranes, blocked with 5% nonfat milk in TBST for 1 h, and incubated overnight at 4°C with primary antibodies (1:1000) (anti‐p‐AKT, anti‐AKT, anti‐Bcl‐2, and anti‐Bax). Membranes were washed, probed with horseradish peroxidase (HRP)–conjugated secondary antibodies (1:10000, 1 h), and visualized using an ECL substrate on a Bio‐Rad chemiluminescence imager. GAPDH (1:10,000) serves as a loading control, and band intensities were quantified using ImageLab software and normalized to GAPDH.

#### 2.2.10. Network Pharmacology and Molecular Docking

The chemical constituents of *Panax ginseng* C.A.Mey., *Ophiopogon japonicus* (Thunb.) Ker Gawl., and *Schisandra chinensis* (Turcz.) Baill. were collected from the TCMSP and BATMAN databases. Compound targets were predicted by the SwissTargetPrediction platform, and intersection targets were obtained by querying the targets with the disease targets of chronic heart failure (CHF) through the OMIM, DisGeNET, and GeneCards databases. Protein–protein interaction (PPI) and topology analyses were performed using the STRING database and Cytoscape software to obtain the core targets, which were imported into the Database for Annotation, Visualization, and Integrated Discovery (DAVID) database for analysis, and Kyoto Encyclopedia of Genes and Genomes (KEGG) pathway enrichment analysis and Gene Ontology (GO) functional enrichment analysis were performed on the common targets using the microbiology platform. Utilize Cytoscape Version 3.10.2 software to construct a network diagram that delineates the intricate relationships between the active ingredients of TCM and their corresponding core targets. Employ degree analysis to separately screen and identify the most effective active ingredient and perform molecular docking of the active ingredient with AKT1 using SwissDock software.

#### 2.2.11. Data Analysis

Statistical significance (*p* value < 0.05) was determined via one‐way ANOVA with Tukey′s post hoc for multigroup comparisons, with results presented as mean ± SEM from more than three independent replicates.

## 3. Results

### 3.1. Network Pharmacology Screening of Core Targets and Molecular Docking

**Figure 1:**
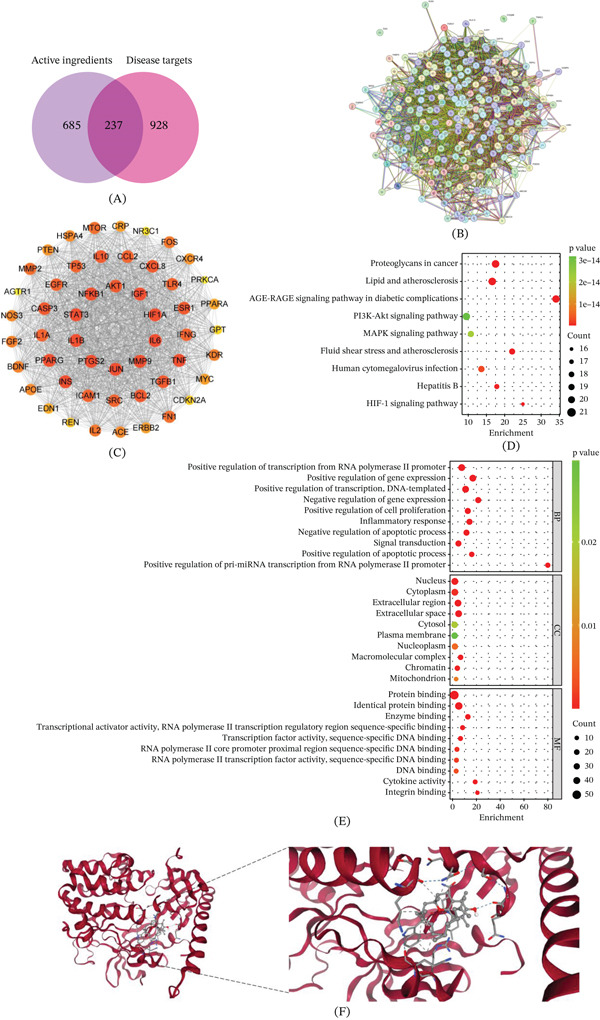
Screening of active ingredients and enrichment analysis. (A) Venn diagram of active ingredient and disease target. (B) PPI network of intersecting genes. (C) Core targets screening and the visualization. (D) KEGG pathway enrichment analysis and (E) GO enrichment analysis. (F) Molecular docking of vitamin E and AKT1.

To explore the therapeutic mechanisms of SMI in CHF, a comprehensive network pharmacology approach was employed. Our analysis identified its active chemical constituents, predicted their putative targets, and integrated them with known CHF‐related genes. We conducted core target screening and pathway enrichment analysis using network pharmacology. The TCMSP and BATMAN databases were used to search for the active ingredients in *Panax ginseng* C.A.Mey., *Ophiopogon japonicus* (Thunb.) Ker Gawl., and *Schisandra chinensis* (Turcz.) Baill., and 1165 active ingredients were obtained. Subsequently, the GeneCards, DisGeNET, and OMIM databases were interrogated under the specific condition of “CHF.” The targets from these three databases were amalgamated, with redundant values removed, leading to a curated list of 922 disease targets. The intersection of 1165 active ingredient targets with the 922 disease targets yielded 237 overlapping genes (Figure 1A). This intersectional analysis provides a strong foundation for exploration of the molecular mechanisms underlying the therapeutic effects of these TCMs in CHF.

We imported 237 intersecting genes into the STRING database and exported the data as TSV files (Figure 1B). Subsequently, these files were loaded into the Cytoscape software to perform network analysis. A total of 52 core targets were identified through this stringent screening process, and a PPI network diagram was constructed for visual representation, as illustrated in Figure 1C. The core targets were imported into the DAVID bioinformatics platform, specifying *Homo sapiens* to ensure the relevance of the analysis. By conducting KEGG pathway analysis, 151 signaling pathways were identified, with the Top 10 selected for further visualization and analysis (Figure 1D). The KEGG analysis revealed significant enrichment in pathways related to cancer pathogenesis, the role of proteoglycans in cancer, lipid metabolism and atherosclerosis, the AGE‐RAGE signaling pathway, the PI3K‐AKT signaling pathway, the MAPK signaling pathway, and the HIF‐1 signaling pathway. These findings underscore the multifaceted roles of active ingredients in modulating key biological processes and pathways, providing a molecular basis for their therapeutic potential in the context of the diseases under investigation. GO enrichment analysis was performed to elucidate the involvement of these targets in various biological processes, molecular functions, and cellular components. The Top 10 results from the GO enrichment analysis were visualized and analyzed using a bioinformatics platform to gain insights into the molecular mechanisms underlying the action of the active ingredients of SMI (Figure 1E). Using Cytoscape 3.10.2, a network diagram was generated to map the complex interactions between active compounds derived from SMI and their core protein targets, as illustrated in Figure S1. This visualization clearly represents the connectivity and molecular interactions between drug constituents and their corresponding targets, offering a holistic perspective on the therapeutic network. Degree analysis was applied to identify vitamin E as the most effective active ingredient. Molecular docking analysis of vitamin E and AKT1, performed using SwissDock (Figure 1F), revealed a favorable binding interaction with a calculated affinity of −6.171 kcal/mol (Table S1). However, we also clarified that vitamin E was identified as a priority through network topology analysis and is more likely to act as a synergistic contributor rather than a dominant compound. In conclusion, we employed a network pharmacology and molecular docking approach to systematically analyze the multitarget pharmacological mechanisms of SMI in CHF. Through cross‐analysis of drug targets and disease genes, a key target was identified. Subsequently, we conducted in‐depth functional analysis to elucidate the core pathways and biological processes mediating the cardioprotective effects of SMI.

### 3.2. SMI Improves Cardiac Dysfunction of MI‐Induced CHF

**Figure 2:**
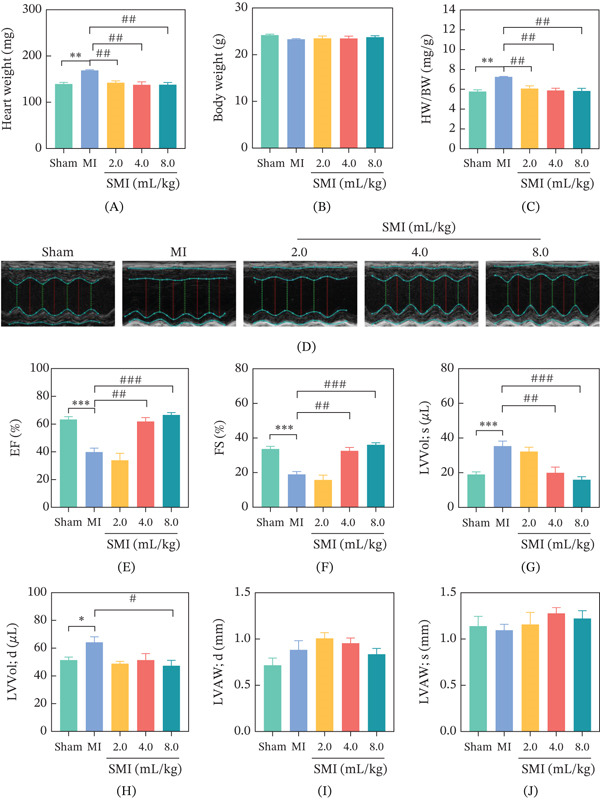
SMI improves cardiac dysfunction of MI‐induced chronic heart failure. The statistical data of (A) heart weight, (B) body weight, and (C) HW/BW. (D) M‐mode echocardiography in mice. (E) Quantified left ventricular ejection fraction (LVEF), (F) left ventricular fractional shortening (LVFS), (G) left ventricular end‐diastolic volume (LV Vol; d), (H) left ventricular end‐systolic volume (LV Vol; s), (I) left ventricular anterior wall‐diastole (LVAW; d), and (J) left ventricular anterior wall—systolic (LVAW; s) of hearts. *n* = 4;  ^∗^ *p* < 0.05,  ^∗∗^ *p* < 0.01,  ^∗∗∗^ *p* < 0.001, ^#^ *p* < 0.05, ^##^ *p* < 0.01, and ^###^ *p* < 0.001.

In an MI‐induced murine model of CHF, SMI was intraperitoneally injected at doses of 2, 4, and 8 mL/kg starting 24 h postsurgery. After 4 weeks of MI, SMI treatment at three doses significantly attenuated the increase in MI‐induced heart weight and heart weight‐to‐body weight ratio (HW/BW) (Figure 2A–C). Echocardiographic assessment further demonstrated that SMI administration markedly ameliorated MI‐induced cardiac dysfunction (Figure 2D and Figure S2). A comprehensive assessment of cardiac function revealed significant impairment in the MI group compared with Sham controls (Figure 2E–J). Echocardiography revealed a marked reduction in LV ejection fraction (EF%: MI 39% vs. Sham 63%, *p* < 0.001) and LV fractional shortening (FS%: MI 19% vs. Sham 33%, *p* < 0.001), along with significant LV dilation, as indicated by increased LV end‐diastolic volume (LV Vol; d: MI 51 *μ*L vs. Sham 64 *μ*L, *p* < 0.01). Chronic administration of SMI resulted in significant functional recovery. The treated animals exhibited substantial improvement in EF and FS, attenuation of LV dilation, and amelioration of diastolic dysfunction. In summary, these data provide evidence that SMI administration effectively ameliorated the impairment of cardiac function caused by MI‐induced CHF, with the magnitude of the benefit exhibiting a clear dose–response relationship.

### 3.3. SMI Inhibits Myocardial Fibrosis and Attenuates Ventricular Remodeling

**Figure 3:**
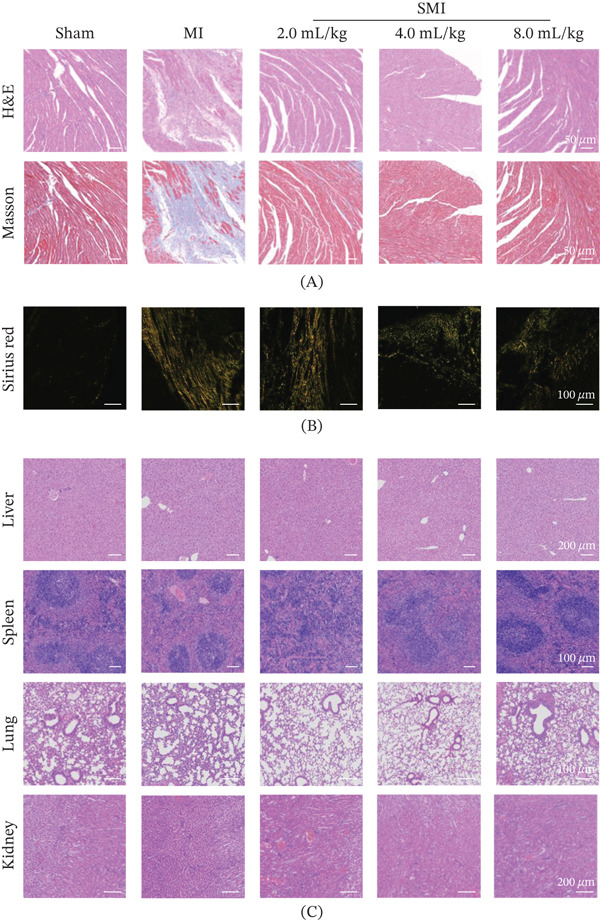
SMI inhibits myocardial fibrosis and attenuates ventricular remodeling. (A) H&E staining and Masson′s trichrome staining of the heart, scale bar = 50 * μ*m. (B) Sirius red staining of the heart, scale bar = 100 * μ*m. (C) H&E staining of the liver, spleen, lung, and kidney, scale bar = 200, 100, 100, and 200 * μ*m.

Subsequently, the cardiac tissue was characterized using H&E and Masson′s trichrome staining to observe the degree of cardiac fibrosis. SMI effectively suppressed MI‐induced cardiomyocyte injury, as indicated by the inhibition of cardiac interstitial fibrosis development (Figure 3A). Polarized light microscopic examination of the cardiac tissue sections stained with picrosirius red revealed distinct patterns of collagen deposition. Increased deposition of birefringent collagen fibers and cross‐linked Type I collagen was prominent within the interstitial spaces and surrounding vasculature in the ventricular myocardium of the MI group compared to that in the Sham group, whereas SMI administration significantly reduced collagen deposition (Figure 3B). Moreover, long‐term administration of different concentrations of SMI has been proven safe. H&E staining of the liver, spleen, lungs, and kidneys showed that SMI administration had no significant effect on the important organs (Figure 3C). In summary, SMI effectively inhibits the development of CHF by suppressing myocardial fibrosis.

### 3.4. SMI Inhibits Myocardial Apoptosis and Regulates Macrophage Polarization In Vivo

**Figure 4:**
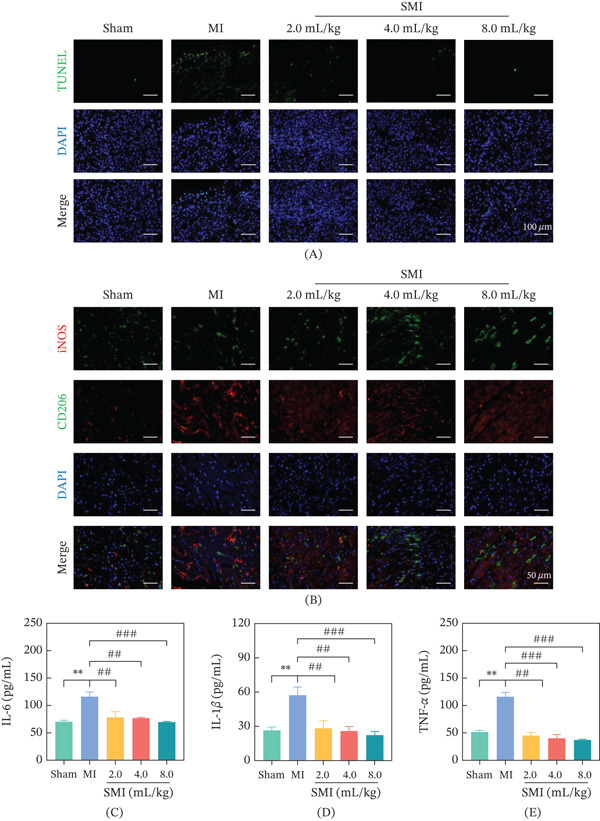
SMI inhibits myocardial apoptosis and regulates macrophage polarization in vivo. (A) TUNEL staining of the heart, scale bar = 100 * μ*m. (B) Double immunofluorescence iNOS/CD206 staining of the heart, scale bar = 50 * μ*m. SMI inhibited MI‐induced serum (C) IL‐6, (D) IL‐1*β*, and (E) TNF‐*α* increase in mice. *n* = 4;  ^∗∗^ *p* < 0.01, ^##^ *p* < 0.01, and ^###^ *p* < 0.001.

TUNEL staining of cardiac tissue sections revealed distinct patterns of DNA fragmentation, indicative of apoptotic cell death. Quantitative analysis showed a significantly increased (*p* < 0.001) proportion of TUNEL‐positive nuclei within the myocardial regions of the MI group compared to that in the Sham group (Figure 4A). TUNEL‐positive nuclei were predominantly localized within cardiomyocytes and, to a lesser extent, interstitial cells in areas showing morphological features of injury, such as inflammatory infiltrates and fibrosis. These findings provide clear histological evidence of heightened cardiomyocyte apoptosis within the affected myocardium of the MI group, suggesting a significant contribution to the observed cardiac dysfunction. Double immunofluorescence staining for the M1 marker iNOS and the M2 marker mannose receptor CD206 was performed to assess macrophage polarization within the MI border zone. Consistent with a proinflammatory milieu, the MI group exhibited significantly elevated iNOS expression in infiltrating macrophages compared to the Sham controls (*p* < 0.001) (Figure 4B). SMI treatment induces a marked shift in macrophage phenotype. In the SMI‐treated MI mice, CD206 signal intensity progressively and significantly increased within macrophage populations across SMI treatment groups, concomitant with a reduction in iNOS expression. This reciprocal modulation of M1 (iNOS^+^) and M2 (CD206^+^) marker expression shows that SMI effectively suppresses the proinflammatory M1 macrophage state and promotes polarization towards the reparative M2 phenotype in the postinfarct myocardium. These findings indicate that SMI facilitates macrophage reprogramming, favoring anti‐inflammatory and tissue remodeling responses after MI. Serum levels of key proinflammatory cytokines (IL‐6, IL‐1*β*, and TNF‐*α*) were quantitatively assessed using enzyme‐linked immunosorbent assay (ELISA) to evaluate the systemic anti‐inflammatory efficacy of SMI treatment in vivo. Compared to the MI group, SMI‐treated mice exhibited a significant reduction in the circulating concentrations of all three cytokines (Figure 4C–E), with the suppressive effect of SMI being dose‐dependent. These data demonstrate that SMI effectively attenuates the systemic inflammatory response in the MI‐induced CHF model, as evidenced by the significant downregulation of IL‐6, IL‐1*β*, and TNF‐*α* in the serum, providing compelling evidence for its potent anti‐inflammatory activity.

### 3.5. SMI Improves Mitochondrial Function of Cardiac Cells Induced by H_2_O_2_ In Vitro

**Figure 5:**
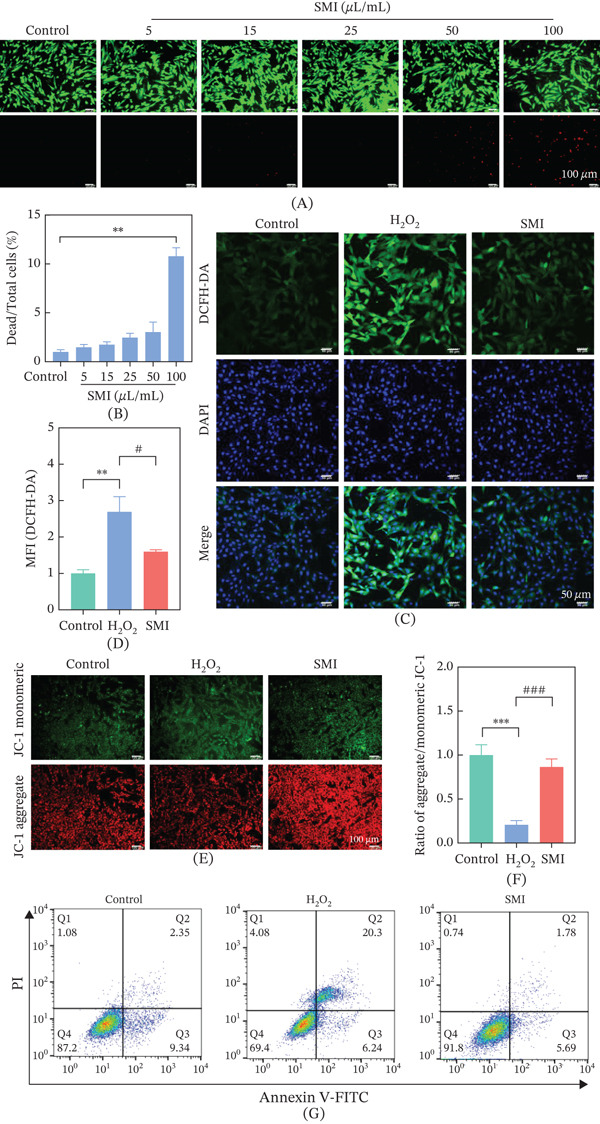
SMI protects cardiomyocytes from H_2_O_2_‐induced injury in vitro. (A, B) Live/dead staining images and statistical chart; *n* = 6,  ^∗∗^ *p* < 0.01 versus control group. (C, D) SMI reduces the increase of ROS induced by H_2_O_2_; *n* = 6,  ^∗∗^ *p* < 0.01 versus control group and ^#^ *p* < 0.05 versus H_2_O_2_ group. (E, F) SMI attenuates H_2_O_2_‐triggered reduction of mitochondrial membrane potential, as measured by JC‐1 fluorescence assay; *n* = 6,  ^∗∗∗^ *p* < 0.001 versus control group and ^###^ *p* < 0.001 versus H_2_O_2_ group. (G) SMI suppresses H_2_O_2_‐triggered apoptotic cell death, as assessed by Annexin V/PI staining.

Subsequently, the effects of SMI on cardiomyocytes and its impact on cell apoptosis in vitro were investigated. Live/dead cell staining and CCK‐8 assays were used to evaluate cell viability. The live/dead cell staining and CCK‐8 assays revealed concentration‐dependent SMI effects on cardiomyocyte viability and cytotoxicity. Low concentrations (≤ 50 *μ*L/mL) showed no significant increase in dead cell counts in calcein‐AM/PI staining (Figure 5A,B) and no reduction in cell viability (Figure S3). In contrast, higher concentrations (100 *μ*L/mL) progressively and significantly decreased live cells with correspondingly elevated PI‐positive cells, indicating substantial cytotoxicity at elevated doses. At therapeutic‐relevant concentrations (25 *μ*L/mL), SMI did not induce cell death, suggesting a safe therapeutic window for further investigation. In H9c2 cardiomyocytes subjected to H_2_O_2_‐induced oxidative stress, SMI treatment (25 *μ*L/mL) reduced intracellular ROS levels by 59% compared to the H_2_O_2_ group (Figure 5C,D). JC‐1 staining revealed that H_2_O_2_‐induced mitochondrial membrane potential (*ΔΨ*m) collapse (red/green fluorescence ratio decreased by 80%) was significantly reversed by SMI (ratio increased by 60% at 25 *μ*L/mL), indicating preservation of mitochondrial integrity (Figure 5E,F). Annexin V‐FITC/PI assays demonstrated that SMI decreased apoptosis rates from 20.3% (H_2_O_2_ group) to 1.78% (25 *μ*L/mL SMI group) (Figure 5G). Collectively, these data confirm that SMI improves H_2_O_2_‐induced mitochondrial dysfunction by mitigating oxidative stress damage in cardiomyocytes, ameliorating mitochondrial membrane potential, and inhibiting apoptotic cell death in vitro, thereby exerting cardioprotective effects.

### 3.6. SMI Attenuates H_2_O_2_‐Induced Cardiomyocyte Injury via AKT Activation and Bcl‐2/Bax Modulation

**Figure 6:**
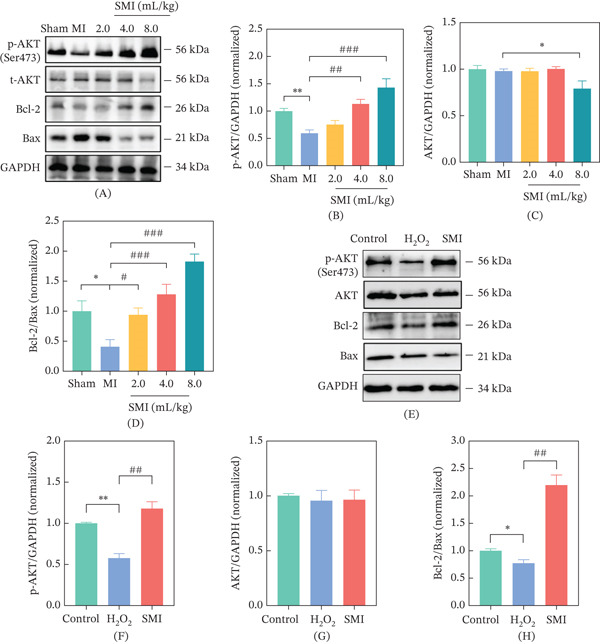
SMI attenuates H_2_O_2_‐induced cardiomyocyte injury via AKT activation and Bcl‐2/Bax modulation. (A) SMI induced the activation of AKT and Bcl‐2 while inhibiting Bax activation in vivo. The statistical results of (B) p‐AKT (Ser473)/GAPDH, (C) AKT/GAPDH, and (D) Bcl‐2/Bax (*n* = 6),  ^∗^ *p* < 0.05 and  ^∗∗^ *p* < 0.01 versus Sham group and ^#^ *p* < 0.05, ^##^ *p* < 0.01, and ^###^ *p* < 0.001 versus MI group. (E) SMI induced the activation of AKT and Bcl‐2 while inhibiting Bax activation in vitro. The statistical results of (F) p‐AKT (Ser473)/GAPDH, (G) AKT/GAPDH, and (H) Bcl‐2/Bax (*n* = 6),  ^∗^ *p* < 0.05 and  ^∗∗^ *p* < 0.01 versus control group and ^##^ *p* < 0.01 versus H_2_O_2_ group.

Western blotting analysis revealed that SMI treatment significantly activated key antiapoptotic signaling pathways in cardiomyocytes. SMI markedly enhanced the phosphorylation of AKT (Ser473) (p‐AKT) compared to the Sham group (*p* < 0.01) (Figure 6A,B), indicating activation of this critical prosurvival kinase, while a high concentration (8 mL/kg) inhibited the expression of total AKT (AKT) (Figure 6C). Concomitantly, SMI administration profoundly upregulated the expression of the antiapoptotic protein Bcl‐2 and significantly downregulated the expression of the proapoptotic protein Bax. This shift in the Bcl‐2/Bax ratio (1.4‐fold) strongly favored cell survival (Figure 6D). Western blotting analysis of H9c2 cells yielded results consistent with the in vivo findings (Figure 6E–H). The coordinated activation of phosphorylated AKT, induction of Bcl‐2, and suppression of Bax provide a molecular mechanism underlying the observed attenuation of cardiomyocyte apoptosis (as evidenced by reduced TUNEL positivity). These findings indicate that SMI exerts its potent cardioprotective and antiapoptotic effects, at least in part, through the AKT‐mediated modulation of the intrinsic mitochondrial apoptosis pathway, specifically by promoting Bcl‐2 expression and inhibiting Bax.

## 4. Discussion

Cardiovascular disease remains the predominant cause of mortality globally, accounting for approximately 28.6% of total global deaths [22]. Impairments in the heart′s structural and functional integrity that compromise ventricular blood filling (diastolic dysfunction) or outflow (systolic dysfunction) can precipitate HF [23]. MI, as a predisposing factor for HF, triggers a cardiomyocyte apoptotic cascade that differs from the initial necrotic cell death in both biochemical pathways and temporal persistence. This apoptotic cascade is distinct from the initial necrotic cell death in its biochemical pathways and temporal persistence. The mitochondrial intrinsic apoptotic pathway is recognized as the dominant mechanism orchestrating this delayed cardiomyocyte loss [24, 25]. This pathway involves a critical balance of proapoptotic and antiapoptotic Bcl‐2 family proteins and mitochondrial outer membrane permeabilization (MOMP) [26]. MOMP facilitates the release of lethal apoptogenic factors, thereby activating effector caspases and executing the apoptotic program, ultimately contributing to the deterioration of cardiac structure and function [27].

This study demonstrated that SMI significantly ameliorated cardiac dysfunction and ventricular remodeling in a mouse model of CHF. We identify the potent inhibition of cardiomyocyte apoptosis via the mitochondrial pathway as the primary underlying mechanism. Our findings align with the established role of persistent apoptosis in post‐MI remodeling. SMI′s efficacy in reducing the number of TUNEL‐positive cardiomyocytes is directly linked to its cardioprotective and antiapoptotic effects. Mechanistically, SMI restored the Bcl‐2/Bax balance, favoring cell survival. The functional benefits (improved EF and FS) and structural improvements (reduced fibrosis and attenuated dilation) were consistent with the preservation of functional cardiomyocytes and the mitigation of apoptosis‐driven inflammatory and fibrotic responses [28, 29]. These results expand previous reports on SMI cardioprotection by providing direct in vivo evidence to prove that it regulates macrophage polarization after chronic MI, improves mitochondrial dysfunction, and clarifies the specific participation of the mitochondrial pathway (Bcl‐2/Bax). In this study, network pharmacology was employed to identify active ingredients from three TCMs constituting SMI. GO analysis revealed that the effective active ingredients are predominantly involved in biological processes such as the inflammatory response and positive regulation of the apoptotic process. They interact with cellular components, including the nucleus, cytoplasm, extracellular region, and mitochondrion, and influence key molecular functions like protein binding, enzyme binding, and cytokine activity. This indicates that the treatment of CHF with SMI encompasses a highly complex biological process involving the regulation of the apoptotic process. KEGG analysis indicated significant involvement in pathways such as proteoglycans in cancer, lipids and atherosclerosis, the AGE‐RAGE signaling pathway in diabetic complications, the PI3K‐AKT signaling pathway, the MAPK signaling pathway, fluid shear stress and atherosclerosis, human cytomegalovirus infection, hepatitis B, and the HIF‐1 signaling pathway. The PI3K‐AKT signaling pathway is intricately linked to multiple pathways and plays a crucial role in regulating various signaling transcription factors in the progression of HF, particularly in modulating cell apoptosis and cell survival [30–34]. This signaling nexus is further amplified by AKT‐mediated stabilization of the Bcl‐2 family antiapoptotic protein Bcl‐xL, creating a robust cytoprotective network that enhances cardiomyocyte resilience against ischemic and oxidative damage [35, 36]. This study proposes that SMI may elicit its therapeutic effects by modulating protein AKT1, thereby regulating cell apoptosis, influencing the mitochondrial function of cardiomyocytes, curbing inflammatory responses, and enhancing cardiac function. While individual components of SMI (e.g., ginsenoside Rg1/Rb1) have shown antiapoptotic effects in vitro or in acute ischemia models [37–40], this study highlights the therapeutic potential of the whole formula in CHF development post‐MI, likely through multicomponent synergy. Additionally, we found strong molecular docking between vitamin E and AKT1, suggesting that vitamin E may also play a cardioprotective role. Compared with standard HF drugs targeting the neurohormonal axes, SMI offers a complementary approach by directly targeting cardiomyocyte survival. We will further identify the key active ingredients in SMI that improve mitochondrial function, regulate macrophage polarization, evaluate their impact on cardiac function and therapeutic efficacy, and conduct carefully designed clinical trials to confirm the efficacy and safety of SMI in post‐MI patients. Several limitations of this study should be acknowledged regarding the translational relevance of the dosing route. SMI is administered intravenously in clinical practice, whereas we used the ip route for repeated daily dosing in mice. The ip route was selected because it is well tolerated in rodents, allows consistent systemic exposure, and has been widely employed in preclinical studies of SMI [41]. The doses (2, 4, and 8 mL/kg) were converted from the clinically used human dose (approximately 0.6–1.0 mL/kg/day for a 60 kg adult) based on body surface area normalization. However, we did not perform a direct pharmacokinetic comparison between ip and intravenous (iv) administration. Therefore, the absolute bioavailability of SMI constituents via the ip route and the degree to which ip dosing mimics the plasma exposure profile of clinical iv infusion remain unclear. This limitation should be taken into account when translating our preclinical efficacy findings to clinical settings. Future studies incorporating iv administration or pharmacokinetic–pharmacodynamic modeling are warranted to address this issue. In conclusion, this study establishes a research framework that integrates TCM empirical therapy with modern computational pharmacology and experimental verification, providing innovative insights for the modernization of TCM research and effectively promoting the transition from experience‐based medicine to evidence‐based medicine. These findings not only provide a solid scientific basis for the clinical application of SMI but also establish a referential methodological framework for investigating the mechanisms of multicompound TCM formulations based on network pharmacology.

## 5. Conclusion

Based on the findings of this study, we draw the following key conclusions: Utilizing network pharmacology approaches, we systematically analyzed its multicomponent synergistic characteristics and predicted the potential molecular mechanisms by which its primary active components act on HF‐related signaling pathways. The establishment of a post‐MI HF animal model confirmed that SMI significantly improves cardiac function parameters, suppresses pathological ventricular remodeling, inhibits macrophage polarization, and reduces the extent of myocardial fibrosis. At the cellular and molecular level, the study validated that SMI modulates the Bcl‐2/Bax balance and inhibits the expression of key proteins in the mitochondrial apoptotic pathway, thereby reducing cardiomyocyte apoptosis and exerting a clear cardioprotective effect. Furthermore, by integrating experimental validation and computational simulations, this study multidimensionally demonstrates that SMI exerts its therapeutic benefits through a “multicomponent, multitarget, multipathway” synergistic mode of action. Additionally, this study establishes a research framework that integrates TCM empirical therapy with modern computational pharmacology and experimental verification, providing innovative insights for the modernization of TCM research and effectively promoting the transition from experience‐based medicine to evidence‐based medicine. These findings not only provide a solid scientific basis for the clinical application of SMI but also establish a referential methodological framework for investigating the mechanisms of multicompound TCM formulations based on network pharmacology.

**Nomenclature**
SMI: Shengmai injection
HF: heart failure
MI: myocardial infarction
LVEF: left ventricular ejection fraction
LVFS: left ventricular fractional shortening
BSA: bovine serum albumin
CHF: chronic heart failure
GO: Gene Ontology
KEGG: Kyoto Encyclopedia of Genes and Genomes
PPI: protein–protein interaction

## Author Contributions

**Nan Hu:** methodology, investigation, data curation, conceptualization, validation, writing – original draft, writing – review and editing. **Hongnv Zhang:** methodology, data curation. **Zhenghao Wang:** methodology, data curation. **Shaodong Zhai:** supervision, resources, writing – review and editing. **Ruiping Zhang:** resources, writing – review and editing.

## Funding

This study was funded by the National Key Research and Development Program of China (10.13039/501100012166) (2023YFC3402800), the National Natural Science Foundation of China (10.13039/501100001809) (32201165, U24A6012, and 82120108016), the Shanxi Provincial Administration of Traditional Chinese Medicine (2024ZYY2C032), the Fundamental Research Program of Shanxi Province (202403021221236), the Research and Innovation Team Project for Scientific Breakthroughs at Shanxi Bethune Hospital (2024ZHANCHI09), and the Key Laboratory of Nano‐Imaging and Drug‐Loaded Preparation of Shanxi Province (202104010910010).

## Ethics Statement

Ethical approval was granted by the Ethics Committee of Shanxi Bethune Hospital (SBQKL‐2021‐040).

## Conflicts of Interest

The authors declare no conflicts of interest.

## Supporting information


**Supporting Information** Additional supporting information can be found online in the Supporting Information section. This file contains all supporting information figures (Figures S1, S2, and S3) and the supporting information table (Table S1). Figure S1 shows the network diagram of traditional Chinese medicine–active ingredients–core targets. Figure S2 presents additional data of echocardiographic assessment. Figure S3 provides the CCK‐8 assays for cellular viability. Table S1 shows the SwissDock of vitamin E and AKT1 (8UW7).

## Data Availability

The raw data supporting the conclusions of this article will be made available by the authors, without undue reservation. All raw data and codes are available upon request.
