# Supplementary material for: Integrated Network Pharmacology and Experimental Validation Approach to Systematically Elucidate the Cardioprotective Mechanisms of Shengmai Injection
Source: Cardiovasc Ther. 2026 Jun 28;2026:6915907. doi: 10.1155/cdr/6915907 (PMC13311316; doi:10.1155/cdr/6915907)
Supplement: Supplementary file 1 — Supporting Information Additional supporting information can be found online in the Supporting Information section. This file contains all supporting information figures (Figures S1, S2, and S3) and the supporting information table (Table S1). Figure S1 shows the network diagram of traditional Chinese medicine–active ingredients–core targets. Figure S2 presents additional data of echocardiographic assessment. Figure S3 provides the CCK‐8 assays for cellular viability. Table S1 shows the SwissDock of vitamin E and AKT1 (8UW7). [file CDR-2026-6915907-s001.docx]

**Integrated Network Pharmacology and Experimental Validation Approach to Systematically Elucidate the Cardioprotective Mechanisms of Shengmai Injection**

Nan Hu^1,*^, Hongnv Zhang^2^, Zhenghao Wang^2^, Shaodong Zhai^1,*^, Ruiping Zhang^3,*^

*1.* *Third Hospital of Shanxi Medical University,* *Shanxi Bethune Hospital, Shanxi Academy of Medical Sciences, Tongji Shanxi Hospital, Taiyuan, 030032, China;*

*2. Institute of Medical Technology Research,* *Shanxi Medical University, Taiyuan, 030001, China;*

*3. The Radiology Department of Shanxi Provincial People’s Hospital Affiliated to Shanxi Medical University, Taiyuan, 030001, China;*

****Corresponding authors:***

Nan Hu

*Third Hospital of Shanxi Medical University, Shanxi Bethune Hospital, Shanxi Academy of Medical Sciences, Tongji Shanxi Hospital, Taiyuan, China*. *Email: hunan@sxbqeh.com.cn*

Shaodong Zhai

*Third Hospital of Shanxi Medical University, Shanxi Bethune Hospital, Shanxi Academy of Medical Sciences, Tongji Shanxi Hospital, Taiyuan, China*. *Email: zhaishaodong@sxbqeh.com.cn*

Ruiping Zhang

*The Radiology Department of Shanxi Provincial People’s Hospital Affiliated to Shanxi Medical University, Taiyuan, 030001, China.* *Email: zrp_7142@sxmu.edu.cn*

***Other author's email:***

*Hongnv Zhang, 3408247105@qq.com*

*Zhenghao Wang, 563641347@qq.com*


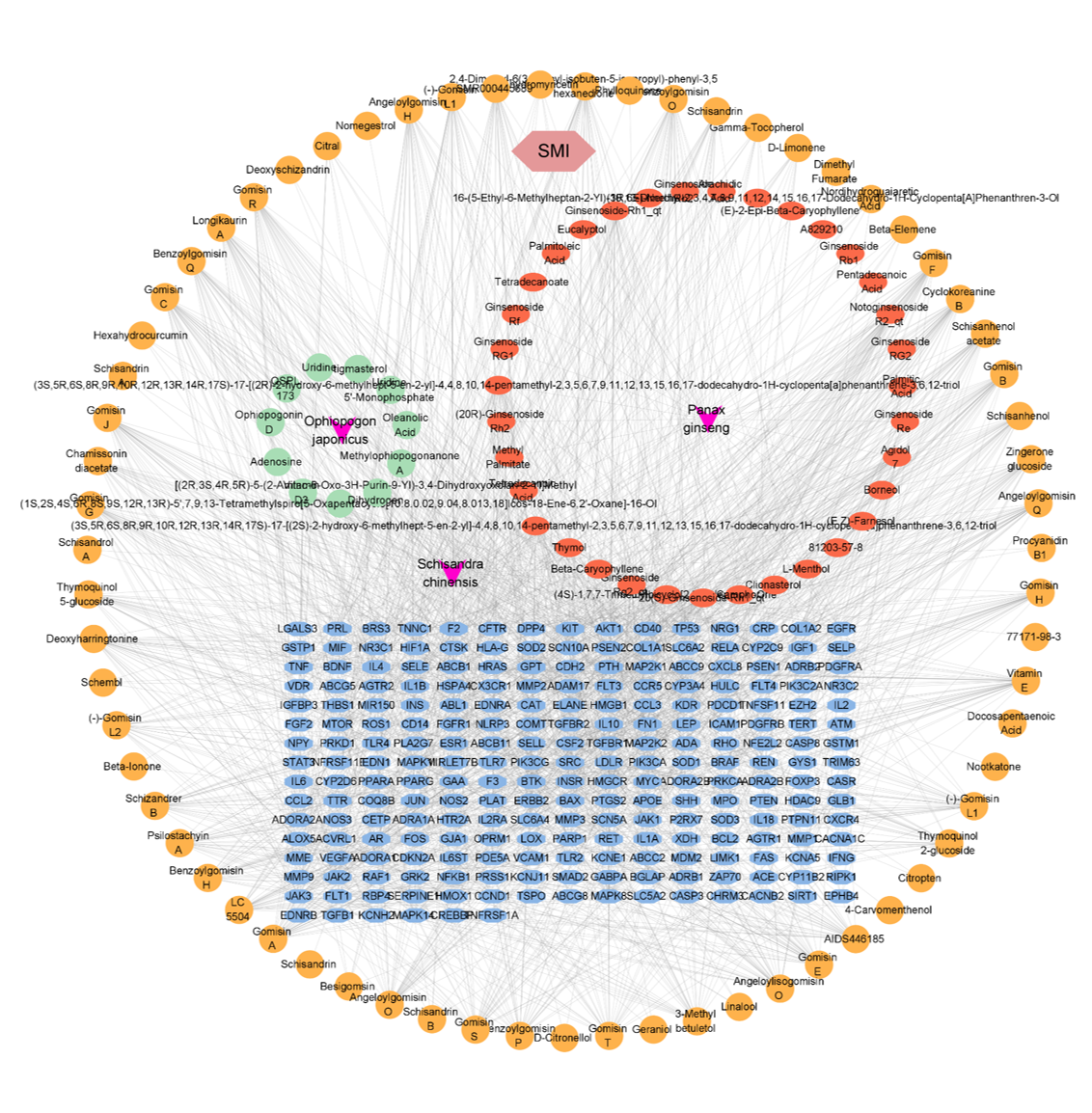


**Figure S1.** Network diagram of traditional Chinese medicine - active ingredients - core targets.


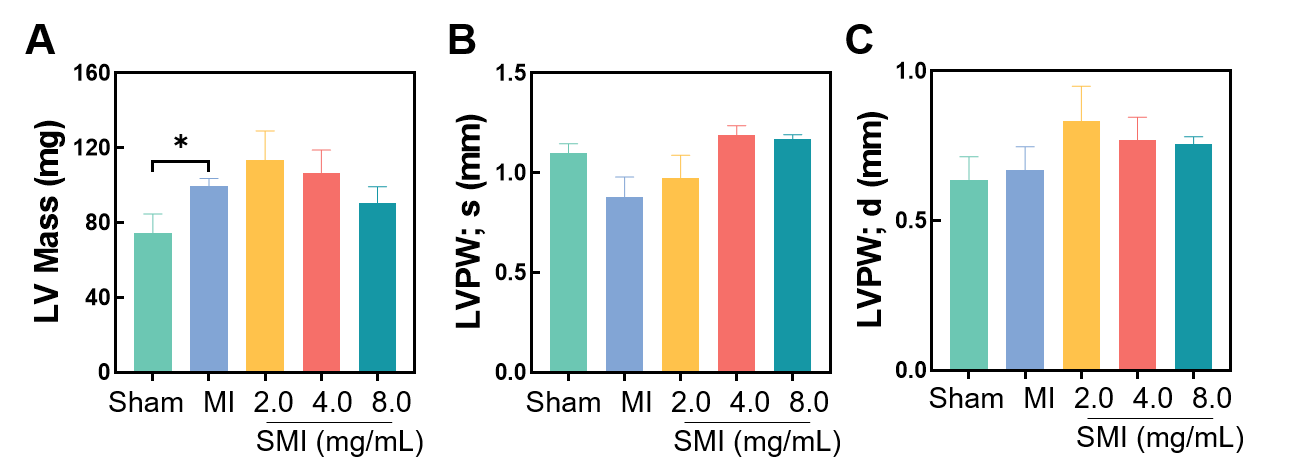


**Figure S2.** Quantified (A) left ventricular Mass (LV Mass), (B) left ventricular posterior wall-systolic (LVPW;s), (C) left ventricular posterior wall-diastole (LVPW;d) of hearts. n=4, *p < 0.05.

**
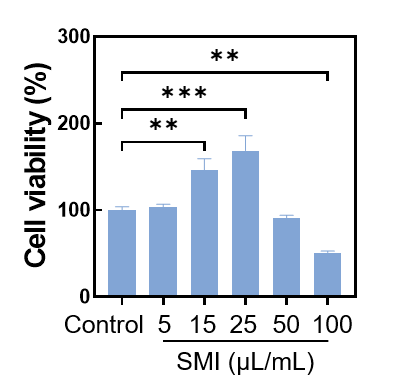
**

**Figure S3.** Cytotoxicity of SMI with concentration of 0-100 μL/mL for 24 h; **p <0.01, ***p < 0.001.

**Table S1**. Swissdock of Vitamin E and AKT1 (8UW7).

| Vitamin E and AKT1 | Calculated affinity (kcal/mol) |
| --- | --- |
| 1 | -6.171 |
| 2 | -6.126 |
| 3 | -6.029 |
| 4 | -5.655 |
| 5 | -5.092 |
| 6 | -4.93 |
| 7 | -4.62 |
| 8 | -4.109 |
| 9 | -3.903 |
| 10 | -3.832 |
| 11 | -3.739 |

|  |
| --- |
